# Nurse-led telecoaching of people with type 2 diabetes in primary care: rationale, design and baseline data of a randomized controlled trial

**DOI:** 10.1186/1471-2296-15-24

**Published:** 2014-02-04

**Authors:** Irina Odnoletkova, Geert Goderis, Frank Nobels, Bert Aertgeerts, Lieven Annemans, Dirk Ramaekers

**Affiliations:** 1 University of Leuven, Kapucijnenvoer 33, Leuven B-3000, Belgium; 2 OLV Hospital Aalst, Aalst, Belgium; 3 Ghent University, Ghent, Belgium; 4 Free University of Brussels, Brussels, Belgium

**Keywords:** Type 2 diabetes mellitus, Telenursing, RCT, Economic analysis

## Abstract

**Background:**

Despite the efforts of the healthcare community to improve the quality of diabetes care, about 50% of people with type 2 diabetes do not reach their treatment targets, increasing the risk of future micro-and macro-vascular complications. Diabetes self-management education has been shown to contribute to better disease control. However, it is not known which strategies involving educational programs are cost-effective. Telehealth applications might support chronic disease management. Transferability of successful distant patient self-management support programs to the Belgian setting needs to be confirmed by studies of a high methodological quality. “The COACH Program” was developed in Australia as target driven educational telephone delivered intervention to support people with different chronic conditions. It proved to be effective in patients with coronary heart disease after hospitalization. Clinical and cost-effectiveness of The COACH Program in people with type 2 diabetes in Belgium needs to be assessed.

**Methods/Design:**

Randomized controlled trial in patients with type 2 diabetes. Patients were selected based on their medication consumption data and were recruited by their sickness fund. They were randomized to receive either usual care plus “The COACH Program” or usual care alone. The study will assess the difference in outcomes between groups. The primary outcome measure is the level of HbA1c. The secondary outcomes are: Total Cholesterol, LDL-Cholesterol, HDL-Cholesterol, Triglycerides, Blood Pressure, body mass index, smoking status; proportion of people at target for HbA1c, LDL-Cholesterol and Blood Pressure; self-perceived health status, diabetes-specific emotional distress and satisfaction with diabetes care. The follow-up period is 18 months. Within-trial and modeled cost-utility analyses, to project effects over life-time horizon beyond the trial duration, will be undertaken from the perspective of the health care system if the intervention is effective.

**Discussion:**

The study will enhance our understanding of the potential of telehealth in diabetes management in Belgium. Research on the clinical effectiveness and the cost-effectiveness is essential to support policy makers in future reimbursement and implementation decisions.

**Trial registration:**

Belgian number: B322201213625. ClinicalTrials.gov Identifier: NCT01612520

## Background

The increasing prevalence of type 2 diabetes poses a challenge to health care systems. Type 2 diabetes is a complex illness that requires continuing medical care and patient self-management education to reduce the risk of long-term complications [1]. Despite the efforts of the healthcare community to improve the quality of diabetes care, about 50% of the population with type 2 diabetes do not reach the guideline recommended treatment targets [1,2]. A recent comparative research of quality improvement strategies in diabetes care found that interventions targeting the system of chronic disease management along with the patient are likely to be more beneficial than those strategies targeting solely health-care professionals [3].

Patient education in disease self-management is commonly recognized as an essential part of diabetes treatment. It has been shown to improve glycaemic control, whereby the intensity of the educational program is believed to be an important predictor of the outcomes [4-6]. However in Belgium, the majority of people with type 2 diabetes are not offered coverage of educational programs. Current evidence of the cost-effectiveness (CE) of diabetes education is limited due to scarcity of publications in this area and the poor quality of the studies [7-9].

A variety of strategies and techniques can be used to provide adequate education in development of problem-solving skills in diabetes management. Offered in a group or individually [10,11]; face-to face or on distance [12-15]; led by people with or without special professional training [16,17]; and depending on the curriculum, - educational programs may demonstrate different results in terms of the clinical and cost-effectiveness. The complexity of these interventions make it difficult to detect the direct effect of specific features of patient education on the outcomes [18,19]. Since a commonly accepted reporting methodology for interventions in prevention and health promotion within clinical trials is lacking, patient education programs are frequently poorly described and difficult to reproduce in other settings.

“The COACH Program” is a well-established target-driven telephone intervention delivered by nurses or dieticians who undergo special additional training [20]. It showed to effectively reduce the disease related risk factors in patients with established coronary heart disease after hospitalization [21,22]. After a research phase, The COACH Program became operational in Australia and extended its curriculum to ten different chronic conditions including type 2 diabetes, in the past 15 years. The clinical and cost-effectiveness of The COACH Program has not yet been tested in Europe.

The specific aims of the study are: 1) to assess whether The COACH Program can be offered by a sickness fund and delivered in cooperation with caregivers in Belgium; 2) to investigate whether The COACH Program helps people with type 2 diabetes to achieve better glycemic control and improved modifiable diabetes risk factors and self-perceived health compared with usual care alone; 3) to analyze the cost-effectiveness of The COACH Program from the perspective of the health care system based on the trial results extrapolated to a life-long horizon.

## Methods/Design

### Study design

The study is a parallel-group RCT, in which patients with type 2 diabetes, affiliated to the Belgian sickness fund Partena, were selected based on their medication consumption data, recruited by their sickness fund, and randomized to receive usual care plus The COACH Program or the usual care alone. The study will assess the difference in outcomes between the two groups. The primary outcome measure is the level of glycohemoglobin HbA1c at 6 months after randomization. The secondary outcomes are: total cholesterol (TC), low-density lipoprotein (LDL), high-density lipoprotein (HDL), triglycerides (TG), blood pressure (BP), Body Mass Index (BMI), smoking status; proportion of people at target for HbA1c, LDL-Cholesterol and Blood Pressure; self-perceived health status, diabetes-specific emotional distress, and satisfaction with diabetes care. The follow-up period is 18 months. All outcome measurements will be collected 3 times: before randomization, upon the graduation from the program (6 months after the program start); and at the end of the follow-up period (18 months after randomization). The allocation ratio is 1:1. Within-trial and modeled cost-utility analyses, - to project effects over a longer time horizon beyond the trial duration, - will be undertaken from the perspective of the health care system if the intervention is effective.

### Study participants

Study participants are adults between 18 and 75 years old with a diagnosis of type 2 diabetes taking glycaemia lowering oral and/or injectable medications. Exclusion criteria are people on corticoid therapy and/or with a debilitating coexisting medical condition such as dialysis, mental illness, cancer; residents of long term care facilities; pregnant women; and people incapable of telephone communication in Dutch.

### Coaching intervention

The COACH Program consists of 5 monthly telephone sessions of 30 minutes on average delivered by certified diabetes nurse educators (further referred to as “coach”). Prior to the intervention, they undergo 5-days training in up-to date clinical guidelines on diabetes self-management and how to give patients the motivation and the skill to improve their risk factors. Coaches are also trained in the use of the COACH Program software for patient administration. Coaches use patient’s baseline data obtained during the first home visit, to assess the individual risk profile and to suggest targets for diabetes risk factors based on the Flemish and international guidelines for good practice in diabetes care [1,23-25]. The therapeutic goals are discussed with the GP by phone before the first coaching session.

Prior to the beginning of the coaching, patients of the intervention group receive a welcome package with information about the program, a book with advice on nutrition in diabetes and a waist circumference meter with a BMI calculator. Patients with HbA1c above 6% (42 mmol/mol) at baseline, who are not in possession of an insurance-covered meter for self-monitoring of blood glucose (SMBG), receive a SMBG set including lancets and strips.

All risk factors associated with diabetes are addressed by the coaches, - glycaemia, lipids, blood pressure, kidney, foot and eye checks, nutrition, physical activity, smoking and alcohol consumption. Patients are instructed on how to perform SMBG and interpret the results. A measurement frequency of one or two day profiles a week is advised. Depending on the type of the diabetes medication – causing hypoglycemia or not – a scheme of four or two times a day respectively is recommended. Considering special skills needed to assist people in smoking cessation, smokers of the intervention group are motivated to contact the tobaccological service of the Belgian Cancer Federation “Tabakstop” that offers tailor-made telephone sessions and is free for all patients.

The coach registers and monitors the biomedical risk factors, the lifestyle/behavioral parameters and the use of the recommended medications. The COACH Program software supports advice on individual treatment targets and the frequencies for the diabetes risk factors control. The software also helps to quickly generate a written coaching report with actual advice and comparison of the current status of the risk factors against the individual treatment targets. These reports are sent to the patients and copied to their GP’s by e-mail or post. The COACH Program software is built upon several databases, such as reimbursed medications and standard comments for each diabetes risk factor.

The COACH Program trains patients to ‘drive’ the process of achieving and maintaining the target levels for their risk factors while working in association with their GP. Coaching is focused on eliminating the knowledge and treatment gap and motivating the patient to apply the appropriate lifestyle and medical therapy. Each session is used as the foundation for the next contact. The coaching model is a continuous five-stage coaching cycle: stage 1 - finding out what the patient knows; stage 2 - telling the patient what he/she should know; stage 3 - assertiveness training; stage 4 - setting an action plan; stage 5 - reassessment at the next coaching session (monitoring). Patients are invited to contact their coach between coaching sessions for questions and further information if required.

### The control group

The control group receives usual care alone. In Belgium, patients on oral glycaemia lowering medications are predominantly treated by their GPs. When insulin therapy needs to be initiated patients become entitled to a “diabetes care trajectory” reimbursed by the national health insurance. The care trajectory is initiated by the GP and implies coordinated care, including diabetes education by a certified diabetes educator, and a yearly contact with an endocrinologist, in addition to the regular GP visits. Patients with advanced diabetes, in need of three or more insulin injections per day, are normally treated in a hospital setting by an endocrinologist, with support of a multidisciplinary team.

All study participants, including the control group, receive a DVD with educational material on type 2 diabetes, its complications and lifestyle recommendations. The laboratory results of the blood analysis are mailed to all study participants and their GPs after each assessment.

### Patient recruitment and randomization

Patients were selected from the administrative database of the sickness fund “Partena” which belongs to the Group of the Independent Sickness Funds, based on the reimbursement data of glycaemia lowering medications in the past 12 months. Prior to the start of patient recruitment, their GP’s were informed about the study by mail. Selected patients were sent a letter of invitation to participate in the study and invited to express their interest by returning an attached response card. Those candidates who expressed their interest, or have not reacted to the invitation within two weeks, were contacted by phone. A home visit for the baseline assessment was scheduled with those patients who confirmed their participation. The assessment visit was carried out by a nurse not involved in the intervention delivery.

Randomization was performed every 2 weeks on average. To achieve a comparable HbA1c distribution within both groups, patients were stratified based on the baseline level of HbA1c: with HbA1c < 7% (53 mmol/mol), or with HbA1c ≥ 7%. Patients from both strata’s were allocated to the intervention or the control group by a data analyst of the Independent Sickness Funds, further not involved into the study, by using a random number generator programmed in Excel.

### Data collection and analysis

During the assessment visits, the nurses register data as stated in Table 1. Weight is measured by electronic scale, patients wearing light indoor clothing, no shoes. Height is measured in the standing position using a portable anthropometer, feet, knees, buttocks and shoulder blades in contact with the vertical surface, no shoes. Waist circumference is measured with a measuring tape according to the guidelines of the Belgian Association for the Study of Obesity [26]. Blood pressure is taken in a sitting position by using a manual sphygmomanometer. Two measurements are taken, the lower systolic and diastolic measurements will be used in the analysis. The level of toxic carbon monoxide (CO) is measured in parts per million (ppm) by a CO meter in addition to self-reporting smoking status.

**Table 1:** Patient data collection at three assessment moments during the trial

**Patient data**	**Method of collection**	**Baseline (T0)**	**T0 + 6 months**	**T0 + 18 months**
Gender	Sickness funds database	x		
Age	Sickness funds database	x		
Education	Self-reporting	x		
Occupation	Self-reporting	x		
Diabetes 2 since	Self-reporting	x		
Comorbidities:	Self-reporting	x		x
*Cardiovascular:* CHD; heart failure; atherosclerosis; past MI; stroke; TIA				
*Respiratory:* COPD; asthma				
*Other:* hypoglycemia; hypertension; dyslipidemia; Kidney disease; neuropathy; depression				
Family history of type 2 diabetes and cardiovascular disease	Self-reporting	x		
Blood pressure	Home visit test	x	x	x
Height	Home visit test	x		
Weight	Home visit test	x	x	x
BMI	Calculated	x	x	x
Waist circumference	Home visit test	x	x	x
Prescribed medications	Sickness funds database + self- reporting	x	x	x
Smoking	Self-reporting + CO test	x	x	x
Lifestyle: physical activity; alcohol consumption; healthy eating	Self-reporting	x	x	x
Diabetes risk factor knowledge test	Self-reporting	x	x	x
EQ-5D	Self-reporting	x	x	x
PAID	Self-reporting	x	x	x
DTSQ – status version	Self-reporting	x	x	x
HbA1c	Pathology lab	x	x	x
Lipid profile: TC; HDL; LDL; TG	Pathology lab	x	x	x
Satisfaction about the COACH program (Intervention group)	Self-reporting		x	
Costs	Sickness funds database; time& material sheets; contracts	x	x	x

*Abbreviations: CHD* Coronary Heart disease, *MI* Myocardial Infarction, *TIA* Transient Ischemic Attack, *COPD* Chronic Obstructive Pulmonary Disease, *EQ-5D* EuroQol 5 dimension health status questionnaire, *PAID* Problem Areas in Diabetes Questionnaire, *DTSQ* Diabetes Treatment Satisfaction Questionnaire; *HbA1c* glycohemoglobin, *TC* total cholesterol, *HDL* high-density lipoprotein, *LDL* law-density lipoprotein, *TG* triglycerides.

The patients are asked to fill in the EQ-5D 3-L as a generic health status survey, the questionnaire Problem Areas in Diabetes (PAID) that measures the level of diabetes-specific emotional distress, and the Diabetes Treatment Satisfaction Questionnaire (DTSQ). All chosen instruments have a well-established cross-cultural validity. In addition, patients in the intervention group are asked to fill in a specific satisfaction questionnaire about The COACH Program. The blood samples are collected and delivered to the laboratory “Meidina Medische Analysen” contracted for the period of the trial. For the biomedical analyses the following methods are used: HbA1c – by ion exchange chromatography; TC and TG – by enzymatic colorimetric methods; HDL – through neutralization of LDL and VLDL. The LDL is calculated using the Friedewald equation. Table 1 summarizes the protocol of patient data collection during the trial.

### Sample size

Sample size calculation was performed by using the stata software. For the subgroup of people with HbA1c ≥ 7%: assuming the difference of 0.4% between the control and intervention group (7.8% vs 7.4%) [4] and the standard deviation of 0.94% as observed within this subgroup at the baseline data analysis, we would need 232 people in both arms to achieve a power of 0.90 in this subgroup. 46% of the participants represent this group. This means that recruiting 555 people in total would be enough to power our hypothesis for the subgroup of people with HbA1c ≥ 7% at baseline if accounting for up to 10% drop-out.

For the total study population: based on the assumption that the mean difference in HbA1c between two groups will be of 0.3% (7.0% vs 6.7%) [2], and standard deviation of 1.05% - as analyzed at baseline – to achieve a power of 0.90 with alpha being 0.05, we would require 514 subjects to complete the study. Allowing for a dropout rate of up to 10%, the target recruitment number should be 566 patients totally, or 283 patients in each arm [27].

### Monitoring intervention integrity

To keep track on the degree to which The COACH Program is delivered as initially planned and to monitor the compliance of the patients and their GPs to the advice of the coaches, the following measures are foreseen: 1) all written coaching reports are reviewed by IO and the head nurse during the first two months of the intervention and selectively thereafter; 2) several coaching sessions are audio-recorded; 3) the coaches register patient’s and GP’s compliance to their advice, particularly in adjusting medication therapy.

### Cost-utility analysis

Cost-utility analysis will be performed if the clinical study demonstrates a positive difference in clinical end points between two study groups [28,29]. Within-trial and modeled cost-utility analyses will be undertaken from the perspective of the health care system, i.e. taking into account direct health care costs to the system including both the cost for the health insurance as the patient out-of pocket costs [30].

The incremental cost-effectiveness ratio (ICER) will be calculated by using the following equation: ICER = ∆Costs/∆QALYs, where ∆Costs is the difference between the mean total cost in the intervention and the control group and ∆QALY is the area between two curves depicting the evolution of the means in QALYs-utilities over time in the intervention and the control group.

Modeling will be applied for projecting effects observed within the trial over a life-time horizon. The assumptions on the progression of type 2 diabetes depending on the known intermediate surrogate outcomes and the associated health status will be derived from published sources. The model’s outcomes validity will be tested through critical appraisal by experts. Future costs of type 2 diabetes without complications, and costs associated with each fatal or non-fatal diabetes-related complication will be estimated based on the epidemiological data available in Belgium and the database of the Independent Sickness Funds. Future costs will be discounted at 3%, future QALYs gained at 1.5% per annum [30]. ICERs will be calculated at various time horizons (e.g., 2, 5, 10, 20 years) [30].

### Costs of the intervention

The fixed intervention costs consist of the investment into the program development, the administrative and supporting personnel, the software maintenance, the consultancy services, and the overhead. Variable costs are those costs which are directly dependent on the number of patients served by the program and associated with the training of the coaches, the recruitment of patients into the program, the actual coaching and administration time, once-off per patient fee, and production and distribution of the program materials for coaches and patients. All costs will be registered prospectively during the trial based on the individual time and material registration and the contractual prices. Fixed costs will be allocated to patients through dividing the total fixed costs by the number of participants in the intervention group.

### Within trial costs and health utilities analysis

Information on the utilization of healthcare services will be obtained from the database of the Independent Sickness Funds. The health care services include primary care visits, visits to emergency departments, visits to specialists, hospital stays, medications, laboratory tests, imaging techniques, paramedical care and other therapies. Out-of-pocket costs will be derived from the published reimbursement regulations. All cost items described above will be specified and expressed in physical, and in monetary units (Euro’s). As baseline measure of costs in both groups, the costs of health care consumption in the 12 months period prior to the trial will be taken. Diabetes – and non-diabetes related costs will be distinguished. Costs imposed by the study that are not part of the routine practice, such as protocol-driven nurse assessment visits and laboratory tests, will not be included into the cost analysis.

The health utility weights will be derived from the Flemish utility value system based on the EQ-5D scores obtained during the assessments visits [30]. QALYs associated with the future diabetes complications will be derived from published sources. QALYs will be calculated assuming linear interpolation between measurement points and calculating the area under the curve, to give a number of QALY gained per patient over the trial period [29].

### Statistical analysis

The two-tailed unpaired t-test will be used for continuous data and chi-square test for proportions. Where appropriate, regression analysis will be applied to account for baseline differences between the two study groups. The results will be expressed as mean (95% CI) for normal data and median (range) for skewed data. Sensitivity analysis will examine the effect of loss to follow-up on the intervention effect. The analysis will be conducted by intention to treat, which means, all patients will be followed for the full duration of the trial. Exploratory analyses will examine the effect of other factors, such as socio-demographic variables, on the outcome.

ICERs will be calculated for the mean and for the upper and lower confidence levels of marginal costs and utilities. One-way sensitivity analysis will be performed to test the relative contribution of different variables to the uncertainty around ICER and presented by means of a Tornado diagram. Probabilistic sensitivity analysis (Monte Carlo simulation) will be performed for all model inputs. The structural uncertainty will be tested through presenting different model scenarios. The results of this analysis will be presented on the cost-effectiveness plane. Probability of willingness to pay by the Belgian Health Care system will be forecasted and presented as a cost-effectiveness acceptability curve [30].

### Ethical issues

For the patient detection procedure based on the medication consumption data, an approval has been obtained from the Belgian Commission for the protection of Privacy. The study protocol has been approved by the Ethical Committee of the University Hospital of Leuven prior to the beginning of the recruitment. Written informed consent was obtained from all patients before initiation of the study. The study is registered at ClinicalTrials.gov, identifier: NCT01612520.

### Patient recruitment and baseline data

**Figure 1:**
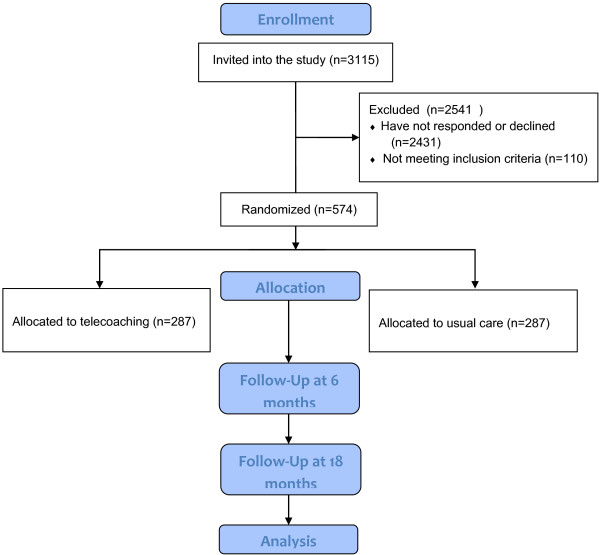
RCT flowchart including the numbers of the enrolled and randomized patients.

Between April 2012 and June 2013, 3115 affiliates of the sickness fund Partena were identified based on the reimbursement data and invited into the study. 685 (22%) agreed to participate. 574 were eligible and took part in the baseline assessment. Figure 1 shows the RCT flowchart including the numbers of enrolled and randomized patients. Table 2 summarizes the baseline characteristics of people allocated to the intervention and control groups.

**Table 2:** Baseline clinical characteristics of patients in the COACH program and usual care groups

**Characteristic**	**The COACH program (n = 287)**	**Usual care (n = 287)**
Sex, no (%)		
Male	173 (60)	180 (63)
Female	114 (40)	107 (37)
Age, years		
Mean (SD)	63.8 (8.7)	62.4 (8.9)
Median (range)	65.9 (35–75)	63.9 (35–75)
HbA1c, % (mmol/mol)	7.0 (53)	7.0 (53)
Mean SD	1.1 (12)	1.0 (11)
Total cholesterol, mg/dl mean (SD)	173 (37)	178 (39)
LDL-C, mg/dl mean (SD)	93 (31)	97 (32)
HDL-C, mg/dl mean (SD)	52 (19)	51 (14)
Triglyceride, mg/dl median (range)	127 (42–1369)	130 (35–1993)
Systolic blood pressure, mmHg mean (SD)	133 (18)	132 (17)
Diastolic blood pressure, mmHg mean (SD)	75 (10)	76 (10)
BMI (kg/m2) mean (SD)	30 (5)	31 (5)
People at treatment target, no (%)		
HbA1c < 7% (53 mmol/mol)	155 (54)	159 (55)
LDL-C < 100 mg/dl	172 (60)	164 (57)
SBP < 140 mmHg	174 (61)	186 (65)
DBP < 80 mmHg	160 (56)	160 (56)
At target for all above risk factors	40 (14)	46 (16)
BMI < 25 kg/m2	33 (11)	28 (10)
In the subgroup with HbA1c ≥ 7% (53 mmol/mol)		
HbA1c, % (mmol/mol)	7.9 (63)	7.8 (62)
MeanSD	1.0 (11)	0.8 (9)
*Self-reported*		
Diagnose type 2 diabetes since, no (%)		
≤ 2 years	46 (16)	41 (14)
≥ 10 years	94 (33)	91 (32)
Smokers, no (%)	40 (14)	54 (19)
People with 1st grade relatives with known type 2 diabetes, no (%)	159 (56)	139 (49)
People with 1st grade relatives with cardiovascular disease diagnosis before 60 y.o., no (%)	93 (33)	90 (32)
With regular hypoglycemia, no (%)	26 (9)	36 (13)
People with other chronic condition(s), no (%)	211 (74)	217 (76)
Coronary heart disease	35 (12)	39 (14)
Atherosclerosis	19 (7)	9 (3)
Heart failure	21 (7)	14 (5)
Past MI	11 (4)	14 (5)
Past stroke	11 (4)	4 (1)
Past TIA	8 (3)	5 (2)
COPD	11 (4)	15 (5)
Asthma	13 (5)	19 (7)
Hypertension	111 (39)	113 (39)
Dyslipidemia	81 (28)	80 (28)
Kidney disease	17 (6)	10 (3)
Depression	19 (7)	24 (8)
Neuropathy	10 (3)	16 (6)
Having physical activity* of 30 minutes at least 5 days per week, no (%)	170 (59)	159 (55)
Having daily healthy diet**, no (%)	181 (63)	157 (55)

*Physical activity is moderate aerobic activity at 50–70% of maximum heart rate (1).

**Healthy diet (HD) means regular meals spread evenly throughout the day, low in fat, particularly saturated fat, and sugar and based on high fiber carbohydrate foods. HD implies daily consumption of at least two pieces of fruit and 300 g vegetables.

## Discussion

Local context can have impact on the acceptability of new forms of care and on their clinical- and cost-effectiveness. Telephone coaching has been applied for patient education in USA and Australia since 1990-ties and has a potential to increase access to health care services. However, in systematic reviews on telehealth in chronic disease management, European studies are poorly represented [13,14,20]. It is therefore important to investigate the transferability of successful telehealth interventions to European countries through studies of a high methodological quality. To increase the added value of such research to patients and policy makers, study designs have to consider the clinical effectiveness, the cost-effectiveness and the implementation potential.

The design of our study has several strengths. 1) The intervention integrity analysis is integrated into the study protocol. The verification of program integrity should be part of the evaluation of any behavioural intervention as lowered adherence to the protocol is often associated with poorer outcome [31]. The integrity assessment also reveals important information about the feasibility of the intervention in real life settings. 2) The COACH Program is well-established in Australia, which enables a comparison of the clinical outcomes in relation to the integrity results. 3) Patients were invited into the study directly, reducing the potential of selection bias. 4) The cost-effectiveness analysis with life-time horizon will help the policy makers to prepare well informed reimbursement decisions. 5) The database of the sickness funds is a reliable and comprehensive source of information on health care consumption.

However, a number of design limitations need to be mentioned. 1) The research setting will to a certain extent affect the study results. Several elements of the protocol are not part of the usual care, e.g. home visits for the purpose of the data collection including the additional laboratory analysis. 2) In the within trial cost-utility analysis, incremental QALYs is one of the primary endpoints of the economic evaluation but can only be derived from a number of generic health surveys with a limited capacity in detecting minor differences in health status, at least in the short term. The disease specific questionnaires have shown to be more sensitive and relevant for use in certain interventions and patient groups. In the last years, researchers have been developing algorithms to translate the scores obtained from disease specific health status questionnaires into health utility weights. However the performance of these algorithms have been criticized [32]. Further research in health-economics should focus on development of cross-cultural instruments capable of capturing common measures of well-being with a higher grade of sensitivity to minor condition divergences.

## Competing interests

The principal investigator (IO) conducts this project as part of her job as employee of the Independent Sickness Funds of Belgium and her PhD at the University of Leuven, Faculty of Biomedical Sciences, Department of Public Health. The authors have no financial interest in this research. The RCT is subsidized by the European Regional Development Fund and the Flemish Government. Other sources of project financing are Partena, MSD and Abbott. The latter two have provided a scientific grant for the clinical trial. The funding sources have no role in the design, conduct, or analysis of the study or in the publication decisions. We thank Mesh and Tabakstop for providing measuring devices for the purpose of the trial.

## Authors’ contributions

IO developed the study protocol and has been managing the research and the intervention implementation. GG critically revised the protocol and the intervention training manuals for coaches and assisted with the statistical analysis. FN critically revised the protocol and assisted with the adaptation of The COACH Program to the Belgian context. BA contributed to the revision of the training manuals for coaches, the design of the protocol and the final approval of the publication draft. LA and DR contributed to the design of the protocol and the final approval of the publication draft. All authors read and approved the final manuscript.

## Pre-publication history

The pre-publication history for this paper can be accessed here:

http://www.biomedcentral.com/1471-2296/15/24/prepub
